# Fruit Nutritional Composition and Seed Reserve Mobilization as Tools for Phenotypic Selection in *Eugenia patrisii* (Myrtaceae)

**DOI:** 10.3390/foods15020188

**Published:** 2026-01-06

**Authors:** Pedro Paulo dos Santos, Elmer Viana Gonçalves, Josiane Celerino de Carvalho, Karen Cristina Pires da Costa, Acacio de Andrade Pacheco, Caris dos Santos Viana, Jaime Paiva Lopes Aguiar, Andreia Varmes Fernandes, Auxiliadora Oliveira Martins, Wagner Luiz Araújo, José Francisco de Carvalho Gonçalves

**Affiliations:** 1 Plant Physiology and Biochemistry Laboratory, National Institute of Amazonian Research—(MCTI-INPA), Manaus 69060-020, AM, Brazil; santospp@gmail.com (P.P.d.S.); elmergoncalves@outlook.com (E.V.G.); carisviana@hotmail.com (C.d.S.V.); varmes@inpa.gov.br (A.V.F.); 2 Bionorte Post Graduate Program (BIONORTE), University of the State of Amazonas, Manaus 69065-020, AM, Brazil; 3 Faculty of Agronomy, Institute of Studies in Agrarian and Regional Development—ISARD Federal University of Southern and Southeastern Pará (UNIFESSPA), Maraba 68500-000, PA, Brazil; karencosta@unifesspa.edu.br; 4 Federal Institute of Education, Science and Technology of Pará, Maraba Rural Campus—(IFPA), Maraba 68502-120, PA, Brazil; acacio.pacheco@ifpa.edu.br; 5 Laboratory of Physical Chemistry of Food National Institute for Amazonian Research—(INPA), Manaus 69060-020, AM, Brazil; jaguiar@inpa.gov.br; 6 Department of Plant Biology, Federal University of Viçosa—(UFV), Viçosa 36570-900, MG, Brazil; auxiliamartins82@gmail.com (A.O.M.); wlaraujo@ufv.br (W.L.A.)

**Keywords:** nutritional status, biochemical markers, Myrtaceae, contrasting phenotypes, domestication, food security

## Abstract

Understanding the integration of metabolic fluxes in fruits and seeds is crucial for identifying key biochemical markers for phenotypic selection in tropical species. This study investigated the Amazonian fruit species *Eugenia patrisii* (Myrtaceae), known for its nutritional and biotechnological potential, to elucidate the link between fruit chemistry and primary reserve mobilization during germination and early seedling growth. Botanical material was collected from an experimental plantation in Maraba, Pará, Brazil. Three contrasting phenotypes (Ph2, Ph3, and Ph6) were analyzed for fruit proximate composition as well as the dynamics of carbohydrates and protein use over seven germination stages. Fruits predominantly contained carbohydrates (76.6–79.3 g/100 g) and proteins (12.7–17.5 g/100 g) and had low lipid content (<5 g/100 g), indicating high energy conversion efficiency. Phenotype Ph6 showed higher protein accumulation and intensive reserve metabolism in late development stages, while Ph2 featured greater soluble sugar content, indicating contrasting reserve allocation strategies. Principal component analysis (PCA) and the indices of integrated metabolic flux (MFI) and total activity (TAI) revealed distinct metabolic cost patterns and biochemical efficiency among phenotypes. Together, these results demonstrate that fruit nutritional attributes and seed metabolic behavior provide quantitative criteria for identifying superior phenotypes, with Ph3 and Ph6 emerging as promising candidates for domestication, breeding, and conservation programs.

## 1. Introduction

Brazil hosts a remarkable diversity of native fruits with distinct sensory properties and high nutritional, economic and functional potential [1,2,3] thereby constituting an important genetic resource for developing novel food products and biotechnological applications. Fruits are widely recognized as essential nutrient sources, playing a key role in health promotion and the prevention of chronic diseases such as cardiovascular diseases, type 2 diabetes, obesity, and cancer [4].

Among the most diverse botanical families is Myrtaceae, predominantly represented by fruit-bearing trees [5,6]. The genus *Eugenia*, one of the most representative within Myrtaceae, includes species of significant economic and ecological value whose edible fruits, timber, and essential oils are commercially exploited and have recognized applications in traditional medicine [7,8]. Many *Eugenia* species, such as *Eugenia uniflora* L., *E. involucrata* DC., *E. jambolana* Lam., *E. pyriformis* Cambess., *E. dysenterica* DC., and *E. patrisii* Vahl., are acknowledged for their nutritional value and volatile compounds [9,10]. Despite existing studies on Amazonian fruits, most remain underexplored.

Accurate identification and taxonomic classification of *Eugenia* species continued to be major challenges for bioprospecting due to high species diversity, morphological overlap, and cryptic species complexes [2,11]. One recent study investigated intra-specific phenotypic variation in seven *E. patrisii* phenotypes during germination based on morphoanatomical and physiological variables [11], highlighting the species potential for cultivation in the Amazon and its importance for food security through the promotion of native fruit consumption and valorization.

*E. patrisii* stands out for its hardiness, adaptation to the Amazon ecosystem, and regionally consumed fruits, which reinforce its relevance in local food security and the bioeconomy. Nevertheless, knowledge gaps persist regarding its physiology and germination of regulatory mechanisms, restricting advances in domestication strategies and its sustainable use.

Nutritional chemical components of *E. patrisii* fruit pulp suggest an alternative food source for Amazonian communities and global commercialization as a food ingredient [8,10,12], similar to other *Eugenia* species which are used as flavorings, supplements and beverage enhancers [1].

Understanding seed chemical composition and reserve deposition/mobilization is fundamental in seed technology, influencing germination, vigor, and storage potential [13,14]. Deepening insights into seed physiological, ecological and biochemical aspects are essential for comprehending the mechanisms that govern establishment and species adaptation in different environments [15].

The efficiency of primary reserve mobilization during germination depends on intrinsic genetic traits and seed physiological quality, determining seedling emergence, vigor, and field adaptation [13,14,15]. In Myrtaceae/*Eugenia*, desiccation sensitivity and carbohydrate, lipid and protein mobilization during germination influence performance and viability [2]. Thus, integrating fruit chemical characterization with the monitoring of seed and seedling reserve mobilization enables a comprehensive understanding of immediate nutritional value and germinative performance, key for genetic improvement and conservation programs of Amazonian fruit trees.

Accordingly, this study aimed to investigate fruit chemical composition and the metabolic dynamics of primary reserve mobilization during germination stages and early growth of contrasting *E. patrisii* phenotypes. Its findings may enhance our understanding of biochemical mechanisms linked to domestication, nutritional valorization and biotechnological potential, contributing to current efforts in food security and functional biodiversity enhancement. We hypothesize that: (1) there is relevant nutritional variation in fruit chemical composition among phenotypes, indicating their potential as functional food resources; (2) there is differential reserve mobilization (carbohydrates and proteins) during germination among contrasting phenotypes reflecting specific metabolic strategies; (3) that these metabolic patterns may identify biochemical markers associated with physiological performance, which are potentially applicable in selecting superior phenotypes for domestication, use, and conservation.

## 2. Materials and Methods

### 2.1. Plant Materials

The botanical material (fruits and seeds) of *Eugenia patrisii* (Myrtaceae) was collected from 21 plants of an agronomic experiment located in Marabá, Pará, Brazil, in November 2022. After collection, the fruits were processed by manually separating the pulp from the seeds. The pulp was allocated for proximate and chemical analyses, while the seeds were stored for reserve mobilization assays during germination.

Initially, orchard plants were categorized into seven distinct phenotypes (Ph1, Ph2, Ph3, Ph4, Ph5, Ph6 and Ph7) based on morphophysiological traits previously described by Santos et al. (2025) [11], including seed biometry, germination performance, and early seedling morphology. From this classification, the most contrasting phenotypes regarding agronomic performance and domestication potential were selected: Ph3 (most promising for cultivation and breeding), Ph2 (intermediate performance) and Ph6 (highest morphological and physiological divergence). These groups are henceforth referred to as phenotypes Ph3, Ph2 and Ph6, respectively.

### 2.2. Proximate Composition of Fruits

Fifty fruits per phenotype were used. Proximate composition assays were conducted on the pulp (exocarp and mesocarp) following standard Association of Official Analytical Chemists [16] methods, covering lipids, proteins, ash, and carbohydrates. Total energy value was estimated using conversion factors of 4 kcal/g for protein or carbohydrates, and 9 kcal/g for lipids.

### 2.3. Germination and Early Seedling Development Stages

**Figure 1:**
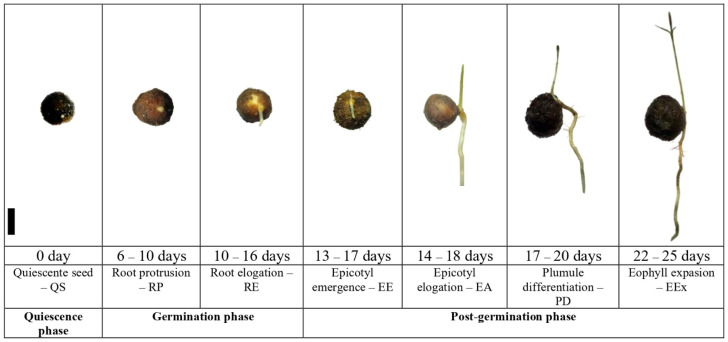
Germination and initial development stages of *Eugenia patrisii* phenotypes. Scale: 1 cm.

Seeds were sown in trays within a greenhouse maintained at an ambient temperature of 35 ± 2 °C at the Laboratory of Plant Physiology and Biochemistry (LFBV-INPA). The substrate comprised fine sand, sterilized by autoclaving at 121 °C and 1 atm for 10 min. Irrigation employed distilled water every 48 h to prevent fungal contamination from water accumulation, or when substrate dryness was observed. Lighting conditions simulated natural light with a 12 h light/dark photoperiod, and tray positions were rotated weekly to ensure uniform environmental exposure. Seeds were considered germinated when radicle protrusion measured between 2 and 5 mm. Germination and early seedling growth were categorized into seven distinct stages as described by Santos et al. (2025) [11]: quiescent seed (QS); radicle protrusion (RP); radicle elongation (RE); epicotyl emergence (EE); epicotyl elongation (EA); plumule differentiation (PD) and eophyll extension (Eex) (Figure 1).

Due to the poorly differentiated morphology of the reserve tissues in *Eugenia patrisii* seeds, the embryo is classified as pseudomonocotyledonous, and the conferruminate cotyledon presents as a single compact structure [11]. Consequently, the entire seed was treated as the embryo since primary reserves are distributed throughout the internal tissue. At each previously defined germination stage, seeds and seedlings were collected, and their attached structures, including tegmen remnants, hypocotyls, radicles, primary and secondary roots, epicotyls and leaves, were carefully removed. This step aimed to ensure that subsequent analyses of primary reserve mobilization were conducted exclusively on the still-active embryonic tissues, enabling precise characterization of metabolic reserve mobilization during early development.

### 2.4. Reserve Metabolism

At each germination stage, 10 seeds were collected, and their metabolic activity immediately arrested in liquid nitrogen. Samples were first stored at −80 °C, subsequently lyophilized using a CHRIST Alpha 1-4 LSC Christ (Osterode am Harz, Germany), ground in an Ika-Werke/M20 analytical mill (Staufen, Germany), and stored again at −80 °C until analysis. Seeds contained approximately 5% of lipid reserves, negating the need for defatting. Biochemical analyses were performed for starch, sucrose, fructose, glucose, and soluble proteins, all extracted and quantified. Carbohydrates were determined enzymatically following Fernie et al. (2001) [17]. Soluble proteins were extracted using TRIS-HCl buffer (mM, pH 7.5) and quantified via the Bradford method (1976) [18] with absorbance measured at 595 nm spectrophotometrically. Amino acids were extracted with 1 M sodium citrate buffer, pH 5.2, supplemented with 0.2% ascorbic acid and measured at 570 nm absorbance [19].

### 2.5. Calculation of Metabolic Indices and Ratios for Primary Reserves

#### 2.5.1. Metabolic Flux Index (MFI)

The metabolic dynamics of transient use and synthesis of reserves over the seven germination and early growth stages were evaluated via the Metabolic Flux Index (MFI), based on an equation determined by Gonçalves et al. (2025) [14]. The MFI was calculated from successive stage variations, SQ–RP, RP–RE, RE–EE, EE–EA, EA–PD, PD–Eex, according to the following equation. Proportional changes in accumulation or degradation of primary reserves (carbohydrates and proteins) between each stage pair, identifying critical metabolic mobilization phases during germination and early growth of *Eugenia patrisii*.

$${MFI}_{i}=\frac{{Value}_{si}-\bar{{Value}_{si-1}}}{\bar{{Value}_{si-1}}}$$

where *MFI_i_* = Metabolic flux index between two stages; ${Value}_{si}$ is the value at stage *i*; and $\;\bar{{Value}_{si-1}}$ is the mean of the replicates at stage *i* − 1.

#### 2.5.2. Net Accumulation Change (NAC)

NAC expresses the metabolic balance between the first germination stage (quiescent seed) and the final early development stage (established seedling), indicating net metabolite accumulation or consumption during the germination process, with responses expressed in the unit of measurement of the respective reserve, described by the following equation. Positive NAC values indicate predominant accumulation or retention, whereas negative values denote predominant consumption or degradation of reserves. NAC calculation followed Gonçalves et al. (2025) [14], quantifying the global metabolic trend of each *E. patrisii* genotype in the seed-to-seedling transition.

$$NAC=E_{f}-{\bar{E}}_{i}$$

where: *NAC =* Net Accumulation Change; $E_{f}$ is the metabolite value at the final stage (Eex); ${\bar{E}}_{i}$ is the mean of metabolite value at the initial stage (QE).

The Total Activity Index (TAI) was calculated by adding the absolute MFI values, representing overall metabolic strategy and cumulative intensity of biochemical transformations of each metabolite during early development, described by the following equation. This index incorporates all absolute transient fluctuations between successive germination stages, interpreting negative changes as positive, thus reflecting total metabolic activity, i.e., the metabolic cost and biochemical associated with primary reserve mobilization and synthesis during germination. The results express the total metabolic cost from quiescence seed to seedling establishment, with calculations based on Gonçalves et al. (2025) [14].

$$TAI=\sum_{i=2}^{n}\left|\frac{{Value}_{si}-\bar{{Value}_{si-1}}}{\bar{{Value}_{si-1}}}\right|$$

where *TAI* = Total Activity Index; ${Value}_{si}$ is the value at stage *i*; $\bar{{Value}_{si-1}}$ is the mean of the replicates at stage *i* − 1 and n = number of germination stages evaluated.

### 2.6. Experimental Design and Statistical Analyses

The experiment was conducted using a completely randomized design under a 3 × 7 factorial scheme. Factors included three phenotypes (Ph2, Ph3, Ph6) and seven germination stages (QS, RP, RE, EE, EA, PD, Eex). For each germination stage and phenotype, ten seeds were collected and pooled to form one composite sample representing that treatment (phenotype × germination stage). From each composite sample, three subsamples were randomly taken and used as analytical replicates for the biochemical assays.

Results were assessed for normality (Shapiro–Wilk) and homogeneity of variance (Levene). Non-compliant data underwent Yeo Johnson transformation [20] followed by reassessment. Reserve contents of all carbohydrates and starch/sucrose metabolic fluxes were transformed, whereas TAI results for fructose and NAC of soluble proteins did not meet assumptions.

Data conforming to assumptions underwent two-way ANOVA (phenotype × germination stage). Significant tests (*p* < 0.05) were followed by Tukey’s HSD post hoc. Non-compliant data were analyzed by nonparametric Kruskal–Wallis and Wilcoxon Mann–Whitney post hoc tests. The categorical heatmap reflecting metabolic intensity and direction from MFI results was constructed per Gonçalves et al. (2025) [14].

Statistical analyses and metabolic, heatmap and principal component analysis (PCA) graphing were performed in R Studio version 2024.12.1+563 [21] using packages of car, agricolae, and FSA for transformations and analyses, and ggplot2 for graphing.

## 3. Results

### 3.1. Nutritional Composition of the Fruits

**Figure 2:**
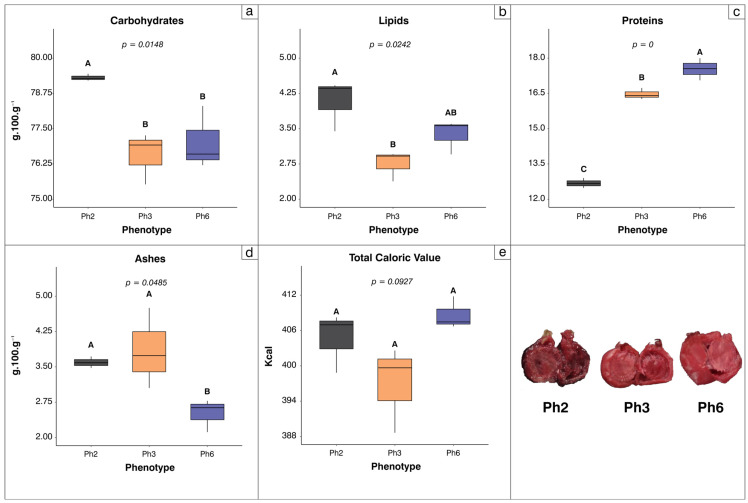
Boxplots of proximate composition of fruits from three contrasting *Eugenia patrisii* phenotypes (Ph2, Ph3, Ph6). Boxes show the interquartile range, lines the median, whiskers non-outlier values, with different uppercase letters indicating significant differences among phenotypes (*p* < 0.05); *n* = 5. The fruit images below illustrate the morphological contrasts among the phenotypes.

The proximate composition analysis revealed consistent variation in the nutritional makeup of the fruit pulps among the evaluated *Eugenia patrisii* phenotypes (Figure 2a–e). It was observed that the major constituents exhibited distinct ranges among the phenotypes, reflecting specific patterns of reserve allocation. These patterns were statistically corroborated by the differentiated distributions of each component, demonstrating that proximate composition is efficient in discriminating phenotypes based on their nutritional profiles. Overall, the evaluated parameters demonstrated significant contrasts in the relative proportions of carbohydrates, proteins, lipids, and ash, indicating unique biochemical profiles for each phenotype.

Carbohydrates constitute the predominant fraction in the fruit’s composition, with contents ranging from 76.6 to 79.3 g per 100 g (Figure 2a). Phenotype Ph2 exhibited a significantly higher carbohydrate content (*p* = 0.015), averaging 79.3 ± 0.1 g 100 g^−1^, while Ph3 and Ph6 showed similar values (*p* > 0.05) of 76.3 ± 0.5 and 77.0 ± 0.6 g.100 g^−1^, respectively, indicating greater availability of soluble sugars at Ph2, associated with the energy supply and sweetness of the fruit.

Lipids were the least abundant reserve in the pulps, with mean values between 2.8 and 4.1 g.100 g^−1^ (Figure 2b). Phenotype Ph2 had the highest lipid content at approximately 4.1 ± 0.3 g.100 g^−1^, followed by Ph6 (3.4 ± 0.2 g.100 g^−1^) and Ph3, which had the lowest value at 2.8 ± 0.2 g.100 g^−1^. The lipid content in Ph2 was significantly greater than Ph3 (*p* = 0.024) but comparable to Ph6 (*p* > 0.05), once again indicating a greater availability of energy and structural precursors in Ph2 fruits.

Proteins were the second most abundant reserve among the three phenotypes, ranging from 12.7 to 17.5 g.100 g^−1^ (Figure 2c). Phenotype Ph6 exhibited the highest protein concentration at 17.5 ± 0.3 g.100 g^−1^, followed by Ph3 at 16.5 ± 0.1 g.100 g^−1^, and Ph2 with the lowest value at 12.7 ± 0.1 g.100 g^−1^. These differences were significant across all phenotypes (*p* = 0), with a proportional difference of approximately 27.4% between the highest and lowest values, which may be associated with structural and enzymatic proteins associated with the onset of ripening.

Average ash contents in the pulps varied between 2.5 and 3.8 g.100 g^−1^ (Figure 2d). Phenotype Ph3 had the highest average ash content at 3.8 ± 0.5 g.100 g^−1^, followed by Ph2 at 3.6 ± 0.1 g.100 g^−1^ and Ph6 with the lowest ash content at approximately 2.5 ± 0.2 g.100 g^−1^. A decreasing gradient in ash content was observed in the order Ph3 > Ph2 > Ph6; however, no significant differences were detected among the phenotypes (*p* > 0.05), indicating that mineral availability among phenotypes remains relatively constant.

Total caloric values of the fruits ranged from 397 to 408.7 kcal per 100 g (Figure 2e). Phenotype Ph6 presented the highest average energy value at 408.7 ± 1.6 kcal.100 g^−1^, followed by Ph2 at 404.7 ± 2.9 kcal.100 g^−1^, and Ph3 with the lowest value at 397.0 ± 4.2 kcal.100 g^−1^. Despite the descending trend, there were no significant differences in caloric content among the phenotypes (*p* > 0.05), suggesting that the energy density of the fruits remains stable across phenotypes, compensating for differences in carbohydrate, lipid and protein fractions.

### 3.2. Mobilization and Metabolism of Carbohydrate Reserves

**Figure 3:**
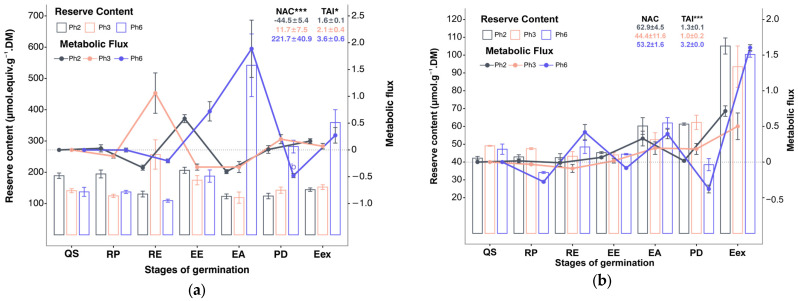

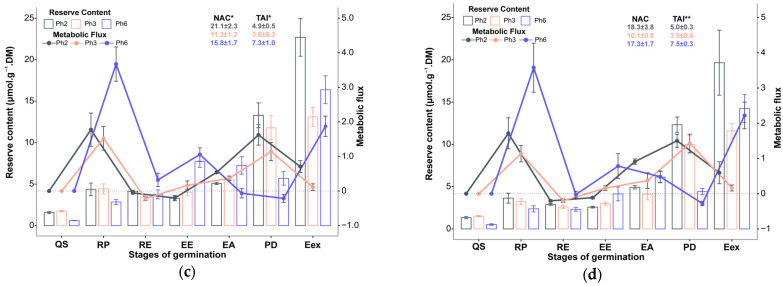
Reserve content and metabolic flux of carbohydrates of three phenotypes of *Eugenia patrisii*, (**a**) = starch, (**b**) = sucrose, (**c**) = fructose, and (**d**) = glucose. QS = quiescent seed; RP = root protrusion; RE = root elongation; EE = epicotyl emergence; EA = epicotyl elongation; PD = plumule differentiation; Eex = eophyll extension, NAC = net accumulation change; TAI = total activity index; * indicates *p*-value ≤ 0.05; ** indicates *p*-value ≤ 0.01; *** indicates *p*-value *≤* 0.001; *n* = 10.

The metabolism of carbohydrates (starch, sucrose, fructose, and glucose) varied throughout the germination stages and exhibited different patterns among the evaluated phenotypes (Figure 3). The following section describes the results related to starch mobilization and the behavior of the main sugars over the seven germination stages of *Eugenia patrisii*.

#### 3.2.1. Starch

The mobilization of starch throughout the seven germination stages differed significantly among phenotypes Ph2, Ph3, and Ph6 (Figure 3a). About reserve content, in the germination phase, starch quantities had a significant reduction during RP to RE in phenotypes Ph2 and Ph6 (*p* < 0.01) while Ph3 increased in the same interval, when compared to quiescence phase (Figure 3a). During EE, there were marked increases (*p* = 0) in starch levels in Ph2 and Ph6, whereas its reserve decreased in Ph3 (Figure 3a). And along EA to Eex, starch content decreased and stabilized for Ph2 and Ph3, while in Ph6 it increased significantly (*p* = 0) in EA and remained; it reduced in PD, and it elevated again in Eex (*p* = 0) (Figure 3a). Among the phenotypes, starch reserves were highest in QS and RP in Ph2 and lowest in Ph6 (*p* < 0.05). In the RE stage, Ph3 exhibited significantly higher starch content (*p* < 0.01) than the other two initial stages. In EA-Eex, starch content was significantly greater (*p* < 0.01) in Ph6.

Regarding metabolism, Ph2 displayed starch degradation along RP to RE, suggesting transient starch synthesis at other stages, notably intense at EE (*p* = 0) (Figure 3a). Ph3 exhibited starch degradation only at RP and EA, significantly at the latter (*p* = 0), with transient synthesis predominant in other stages, especially RE (*p* = 0) (Figure 3a). Ph6 showed more extensive starch degradation in early post-germination (RP and RE) and during PD, with significant degradation at RE (*p* = 0). Transient starch synthesis in Ph6 occurred at EE and EA and at the final stage of Eex, (*p* = 0) (Figure 3a). Metabolic divergences in starch reserves between phenotypes were most notable during early development during RE to PD (Figure 3a).

The metabolic flux balance was positive in Ph3 and Ph6, significantly greater in Ph6 (*p* < 0.001), and negative for Ph2, though statistically similar to Ph3. Total metabolic activity was significantly distinct in Ph3 compared to others (*p* < 0.05), with Ph2 showing the lowest values (Figure 3a). Considering the net accumulation index (NAC), starch showed a negative balance in Ph2, indicating net consumption of this reserve between the quiescent seed and the established seedling, while Ph3 exhibited a slight positive balance and Ph6 showed a markedly positive NAC, reflecting intense net accumulation in the final stage. These results confirm that Ph2 adopts a strategy centered on the direct mobilization of starch to meet energy demand, while Ph6 presents a pattern of intense remobilization followed by starch re-accumulation during seedling establishment. The total activity index (TAI) was lowest in Ph2 and highest in Ph6, indicating that Ph6 experienced the highest metabolic cost associated with starch turnover throughout germination, while Ph2 followed a more linear and energetically conservative degradation pattern.

#### 3.2.2. Soluble Sugars

About reserve content, Ph2 and Ph3 showed minimal fluctuation in sucrose levels from SQ to EE (Figure 3b). After this, sucrose content significantly increased (*p* = 0), stabilizing until PD, then increasing again significantly at the final stage (*p* = 0) (Figure 3b). In Ph6, sucrose levels fluctuated more throughout the germination phase, with the highest significant contents at the last stage (*p* = 0) (Figure 3b).

Fructose contents fluctuated in Ph3 from the quiescence to germination phases, stabilizing at EE and markedly rising during the last three stages (*p* = 0), whereas Ph2 increased in the germinative phase, stabilized until EA, then increased significantly in the last two stages (*p* = 0) (Figure 3c). Ph6 showed gradual fructose rises significantly in quiescence, germination, and post-germination phases (*p* = 0) (Figure 3c). At RP and PD, fructose levels followed Ph3 > Ph2 > Ph6, with Ph6 significantly exceeding the others at EE (*p* < 0.01) (Figure 3b). Glucose showing increasing levels of germination for all (Figure 3d).

Metabolic analysis showed no significant differences in sucrose metabolism between Ph2 and Ph3 stages, except during EA to PD (Figure 3b). Sucrose degradation occurred only at RE in Ph2 and at RP and RE in Ph3. In Ph6, degradation was observed at RP, EE, and PD, (*p* < 0.05), with significant transient synthesis in the last stage (*p* < 0.05) (Figure 3b). Metabolic differences corresponded to opposing sucrose metabolism patterns wherein Ph6 differed from Ph2 and Ph3 (Figure 3b). Total metabolic activity was significantly higher for Ph6 compared to others (*p* = 0) (Figure 3b).

Fructose metabolism revealed sugar degradation in early stages, RE of Ph2 and Ph3, and EE in Ph2 (Figure 3c). Transient synthesis occurred at other stages, particularly intense during RP for both Ph2 and Ph3 (*p* = 0). Ph6 showed degradation in late stages (EA and PD), with intense transient synthesis early in germination (*p* = 0). Compared to equivalent stages, transient syntheses in Ph6 were significantly greater than Ph3 (*p* < 0.01) and, in the last stage, the synthesis intensities ranked Ph6 > Ph2 > Ph3 (*p* < 0.01). All phenotypes had positive metabolic balances, highest in Ph2 (*p* < 0.05), while metabolic activity was significantly greater in Ph3 (*p* < 0.05) (Figure 3c). Glucose metabolism showed no significant differences, similar to sucrose, but with differing metabolic dynamics suggested among phenotypes during the stages (Figure 3d).

Glucose degraded during RE in all phenotypes and additionally in EE in Ph2 and PD in Ph6 (Figure 3d). Significant transient glucose synthesis occurred during RP in all phenotypes and during PD in Ph2 and Ph3 (*p* = 0). Ph6 showed significantly higher total metabolic glucose activity relative to other phenotypes (*p* < 0.01) (Figure 3d).

Soluble carbohydrates showed positive NAC values in all phenotypes, indicating a net accumulation of sucrose, fructose, and glucose from the quiescent seed to the established seedling, with Ph2 and Ph6 generally exhibiting the highest balances. However, TAI values revealed distinct metabolic strategies among the phenotypes, with Ph6 consistently showing the highest metabolic cost activity, reflecting intense turnover and pronounced fluctuations between degradation and synthesis.

### 3.3. Proteins and Amino Acids

**Figure 4:**
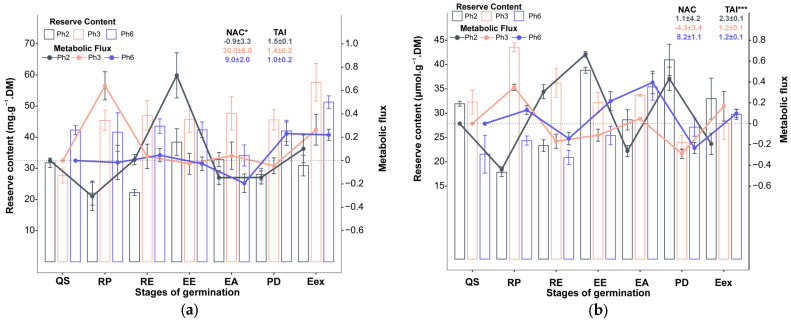
Reserve content and metabolic flux of carbohydrates of three phenotypes of *Eugenia patrisii*, (**a**) = soluble proteins, (**b**) = amino acids. QS = quiescent seed; RP = root protrusion stage; RE = root elongation; EE = epicotyl emergence; EA = epicotyl elongation; PD = plumule differentiation; Eex = eophyll extension; NAC = net accumulation change; TAI = total activity index; * indicates *p*-value ≤ 0.05; *** indicates *p*-value *≤* 0.001; *n* = 10.

The results regarding the levels and metabolism of protein reserves revealed significant differences both among the germination and post-germination phases and between the evaluated phenotypes (Figure 4).

In regard soluble protein reserve contents exhibited minimal significant variation between phenotypes Ph3 and Ph6, with the highest levels recorded in the final stage (*p* < 0.05) (Figure 4a). Ph2 showed fluctuations during germination and pos-germination, reaching its peak concentration at the EE (*p* < 0.05) (Figure 4a). Distinct patterns emerged across germination stages, considering that Ph6 significantly surpassed others in the quiescent seed stage (*p* < 0.01) (Figure 4a). Except during epicotyl stages (EE and EA), Ph2 consistently displayed significantly lower soluble protein contents (*p* < 0.05) (Figure 4a).

The NAC values revealed a net depletion of soluble proteins in Ph2 and positive metabolic balances in Ph3 and Ph6, confirming that Ph2 relied more heavily on protein catabolism throughout germination, whereas Ph3 and Ph6 progressively restored or accumulated protein reserves up to seedling establishment. Although the magnitude of accumulation was greater in Ph3, Ph6 exhibited a more stable positive balance, indicative of a more regulated protein turnover. Accordingly, the TAI values for soluble proteins remained low across all phenotypes, indicating that, despite stage-specific fluctuations between synthesis and degradation, protein metabolism required a relatively modest metabolic cost and biochemical investment during early development.

Amino acid reserve content fluctuated throughout germination and post-germination in Ph2 and Ph3, with the highest levels occurring during RP in Ph3 and PD in Ph2 (*p* < 0.01) (Figure 4b). Ph6 presented alternating fluctuations and stability levels across the stages (Figure 4b). At the beginning of germination, amino acid concentrations ranked Ph2 < Ph6 < Ph3, differing significantly (*p* < 0.01) (Figure 4b). Amino acid metabolism was more dynamic than soluble protein reserves, distinguishing Ph2 from Ph3 and Ph6. Both Ph3 and Ph6 exhibited significant degradation during RE, notably in PD (*p* < 0.01), and EE (only Ph3) (Figure 4b). Transient amino acid synthesis intensified at RP and EA in Ph3 (*p* < 0.01) and EE to others (*p* = 0). No significant differences were noted in final metabolic balance among phenotypes; however, Ph3 showed a negative balance, while Ph2 and Ph6 were positive. Total metabolic activity was significantly higher in Ph2 (*p* = 0) (Figure 4b).

The NAC and TAI indices show that Ph2 operates under a high-metabolic-cost strategy, characterized by intense mobilization and resynthesis of nitrogen compounds. Ph3 depends on an equally intense, but predominantly transient, mobilization, resulting in a net loss of amino acids at the end of the process. While Ph6 adopts a more balanced and energetically efficient metabolic pattern, promoting the progressive stabilization of both soluble proteins and amino acids as the plant establishes itself.

### 3.4. Total Metabolism

**Figure 5:**
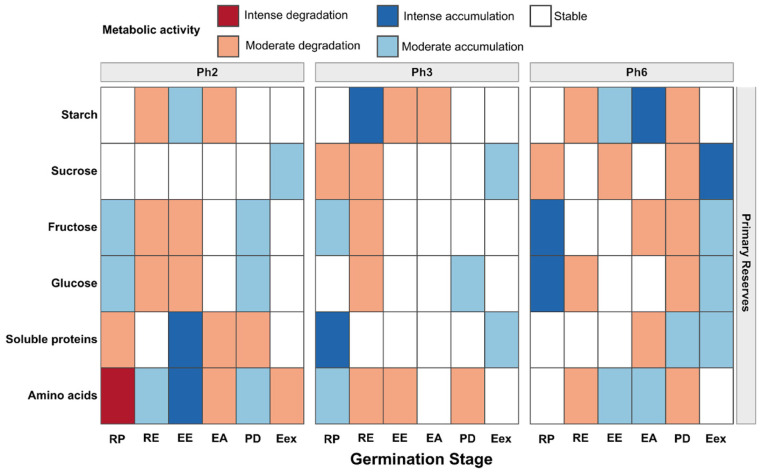
Categorical heatmap of metabolic activity in primary reserve mobilization of *Eugenia patrisii* during germination and post-germination stages. RP = root protrusion; RE = root elongation; EE = epicotyl emergence; EA = epicotyl elongation; PD = plumule differentiation; Eex = eophyll extension.

Considering the overall metabolic context, encompassing all carbohydrates and proteins, distinct patterns of reserve mobilization were observed among phenotypes Ph2, Ph3 and Ph6 throughout the germination stages (Figure 5).

The metabolic patterns observed in the phenotypes were generally moderate regarding degradation, except for amino acids in the RP stage in Ph2. Additionally, Ph2 exhibited intense metabolism related to the accumulation of soluble proteins and amino acids in the EE stage (Figure 5). In Ph2, fructose and glucose metabolism showed similar patterns that were inverse to starch metabolism during early post-germination (RE-EA). Proteins and amino acids showed similar patterns except during PD (Figure 5).

In Ph3, metabolic synthesis intensity was highest in the early germination phase for both soluble proteins and starch, with starch metabolism opposing that of soluble sugars during RE (Figure 5). Moderate amino acid degradation during the post-germinative phase suggested the beginning of the plant’s transition from autotrophic to heterotrophic metabolism (Figure 5).

Ph6 showed high accumulation of simple soluble sugars in the initial stage and more complex sugars in the final stages (Figure 5) and predominance of moderate degradation was observed to final stages, while amino acids demonstrated dynamic metabolic fluctuations in accumulation and degradation, contrasting with soluble proteins from EA onward (Figure 5).

**Figure 6:**
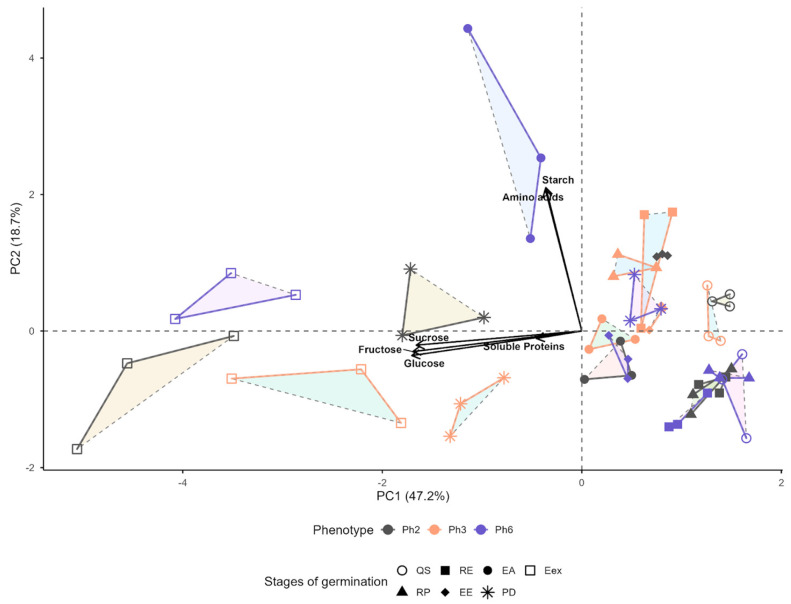
Principal component analysis (PCA) of primary metabolites and minerals in seeds of three *Eugenia patrisii* phenotypes throughout seven germination stages, QS = quiescent seed stage; RP = root protrusion stage; RE = root elongation stage; EE = epicotyl emergence stage; EA = epicotyl elongation stage; PD = plumule differentiation; Eex = eophyll extension.; *n* = 10.

Principal component analysis of primary metabolites and minerals in seeds of three *Eugenia patrisii* phenotypes during seven germination stages revealed clear metabolic reprogramming patterns. The first principal component (PC1, 47.2% of the variance) was mainly associated with soluble carbohydrates (sucrose, fructose and glucose), whereas the second principal component (PC2, 18.7% of the variance) was driven predominantly by starch and free amino acids, indicating a shift from reserve to mobilized carbon and nitrogen pools during germination. Soluble proteins contributed mainly to a subsequent component, suggesting that variation in protein content is important but orthogonal to the main gradients of carbohydrate and amino acid dynamics (Figure 6).

Along the germination trajectory, the phenotypes showed a general transition from starch- and amino-acid–rich quiescent seeds and early stages (QS, RP and RE) towards stages characterized by higher levels of soluble sugars (EE, EA, PD and Eex), consistent with progressive reserve mobilization and seedling establishment. Early stages tended to cluster in regions of higher PC2 and lower PC1 scores, reflecting a predominance of structural carbohydrate and nitrogen reserves, in which later stages shifted to higher PC1 scores as sucrose, fructose and glucose accumulated. This pattern indicates a coordinated conversion of starch into soluble sugars that support embryonic growth and the metabolic demands of developing seedlings.

Despite this common trajectory, the phenotypes differed in the timing and intensity of their metabolic strategies. Ph6 was in regions of the biplot with higher PC2 scores in intermediate stages, it suggests a more conservative strategy characterized by sustained starch and amino acid reserves before extensive sugar accumulation, possibly due to delayed starch hydrolysis and slower nitrogen mobilization, resulting in a more gradual. In contrast, Ph2 and especially Ph3 shifted earlier and more markedly towards higher PC1 scores at advanced stages, indicating a more pronounced and rapid conversion of reserves into soluble carbohydrates, which may underpin differences in germination rate and seedling vigor among phenotypes.

## 4. Discussion

Recent studies on the nutritional composition and proximate analysis of fruits, as well as the mobilization of primary reserves throughout germination in *Eugenia* species, aim to characterize these species and identify biotechnological potentials to support seed technology applications [1,22,23,24]. However, studies on intraspecific variations reflecting phenotypic plasticity remain scarce, which this work seeks to highlight.

### 4.1. Nutritional Composition of the Fruits

The carbohydrate-dominated reserve profile of *E. patrisii* fruits aligns with patterns observed in other *Eugenia* species, where soluble sugars and structural polysaccharides constitute the main energy reserves in mature fruits, including *Eugenia brasiliensis*, *E. stipitata* [1], and *E. uniflora* and *E. pyriformis* [3,25]. In tropical fruit species, carbohydrates play critical roles in fruit ripening, organoleptic quality, and initial metabolic supply to seeds, acting as primary substrates for respiration and biosynthesis during germination [26].

Considering that carbohydrates, such as soluble sugars, particularly sucrose, function as key molecules not only in energy supply but also in the modulation of signaling pathways that govern ripening, pigment accumulation, and sink strength, the high carbohydrate content observed in Ph2 may reflect an enhanced storage capacity or a greater efficiency of phloem unloading. These features could have structural and sensory consequences, such as increased sweetness, during fruit development [27].

The values for the lipid fractions of the studied phenotypes Ph2, Ph3 and Ph6 are consistent with the values reported for other Myrtaceae fruits, such as include *E. brasiliensis*, *E. stipitate* [1], *E. uniflora* and *E. pyriformis* [3,25]. In forest seeds, lipids provide high-energy reserves mobilized via β-oxidation and the glyoxylate cycle, classical mechanisms described by Bewley et al. (2013) [26]. In *Eugenia* species, triacylglycerols and structural phospholipids contribute to energy provision and may influence seed physiological behavior, including desiccation tolerance [24]. Desiccation tolerance may be associated with the role of lipids in maintaining membrane integrity, serving as precursors of volatile compounds, and contributing to energy density. Moreover, the accumulation observed in Ph2 may reflect specific metabolic traits that influence aroma, postharvest stability, or tissue resilience under environmental stress conditions [28].

Proteins constituted the second most prominent reserve fraction among phenotypes, with Ph6 showing the highest protein content. A higher protein content may indicate an increased investment in structural and enzymatic proteins, particularly those involved in cell wall metabolism, which could explain the distinct profile observed in Ph6 [29,30,31,32]. This metabolic pattern corroborates observations in other *Eugenia* species such as *E. brasiliensis*, *E. stipitata* [1], *E. uniflora*, *E. pyriformis* [3,25] and *Eugenia* (*Syzygium paniculatum*) [33].

The average ash contents in *E. patrisii* fruits fall within ranges reported for other *Eugenia* species. For instance, *E. stipitata* presents approximately 2.31 g 100 g^−1^ [1], *E. pyriformis* 4.1 g 100 g^−1^ [3] whereas *E. calycina* and *E. stigmatosa* show lower averages near 0.5 g 100 g^−1^ [4]. These variations may arise from genetic and phenotypic differences among species and environmental factors such as soil type, mineral availability, and fruit maturation stage, all controlled in this study by sampling individuals from the same location and maturity stage. Although statistically non-significant among phenotypes, the trend Ph3 > Ph2 > Ph6 suggests differential mineral absorption and translocation patterns in *E. patrisii*, supporting phenotypic plasticity in mineral accumulation within Myrtaceae species. These minerals may serve as alternative sources for bioactive compounds in *Eugenia* [3].

Energy values observed in *E. patrisii* fruit pulps (397–409 kcal 100 g^−1^) rank among the highest recorded for the genus, reflecting a biochemical composition dominated by carbohydrates and proteins with low lipid content. This caloric density exceeds that reported for *E. stipitata* (280–350 kcal 100 g^−1^) [1], *E. calycina* and *E. stigmatosa* (250–310 kcal 100 g^−1^) [4], and *E. calycina* and *E. stigmatosa* (49–62 kcal 100 g^−1^) [3]. High energy content in *E. patrisii* aligns with its substantial carbohydrate fraction, the main contributor to fruit caloric density, consistent with trends in Amazonian Myrtaceae where soluble sugars and structural compounds represent major carbon reserves [33,34]. This trait grants *E. patrisii* fruits not only high nutritional value but also ecological and functional potential by attracting frugivorous dispersers and providing significant energy for germination and seedling establishment. Biotechnologically, this caloric and nutritional profile reinforces the species’ potential as a regional food resource and as a model for studies linking chemical composition and physiological vigor in tropical fruits.

### 4.2. Reserve Mobilization During Germination

#### 4.2.1. Mobilization and Metabolism of Carbohydrate Reserves

Carbohydrate mobilization plays a central role in germination of *Eugenia* species, primarily due to the recalcitrant behavior of their seeds. These seeds maintain high moisture content, supporting intense metabolic activity and rapid energy demand from germination onset. This high metabolic activity is directly linked to desiccation sensitivity, a hallmark of recalcitrant seeds in the genus [2].

In *E. uniflora*, starch and soluble sugars constitute the main reserve fraction, with limited lipid contribution [23]. Similarly, histochemical analyses of *E. stipitata* confirm starch as the predominant reserve in the embryo, highlighting reliance on carbohydrates for initial energy supply [35]. The present study results display marked carbohydrate reserve variations and fluctuations in starch, sucrose, fructose, and glucose contents during germination stages. These findings align with widely described metabolic patterns in *Eugenia* species and reinforce that energy supply for germination is primarily carbohydrate-dependent, with minimal lipid involvement.

Although *E. patrisii* predominantly accumulates carbohydrate reserves, its mobilization patterns follow general metabolic principles as described by Angerman et al. (2024) [36]. These authors demonstrated that the heterotrophic phase simultaneously depends on the energy supply and the nitrogen mobilized from the cotyledons, with a peak in protein transfer occurring during the second week of development. In *E. patrisii*, carbohydrates sustain initial respiration and enzymatic activation, whereas proteins provide essential nitrogen for tissue synthesis and expansion. Thus, despite chemical composition differences, the physiological model proposed by Angermann et al. (2024) [36] supports the notion that germination requires the integration of energy and nitrogen sources, offering a solid comparative framework to interpret the differential roles of carbohydrates and proteins in the evaluated phenotypes.

Previous studies have reported general carbohydrate fluctuations during germination in *Eugenia* species such as *E. uniflora* and *E. astringens* [2,3]. However, our results for *E. patrisii* reveal a more complex picture, with three analyzed phenotypes showing contrasting intraspecific metabolic strategies involving significant variations in starch mobilization and soluble sugar redistribution in germination stages. As highlighted by Pontes et al. (2002) [37], the levels of starch and soluble sugars present in the seeds and the dynamics of their mobilization throughout germination are species-dependent characteristics. Additionally, carbohydrate consumption can accelerate in the early stages of germination or intensify in later stages, reflecting physiological differences between species. This phenotypic resolution, not previously described for *Eugenia* species, significantly advances our understanding of physiological plasticity and highlights *E. patrisii* phenotypes as promising sources of biochemical markers for selection and breeding programs.

##### Starch

In the initial stages (QS, RP and RE), stability in starch content was observed for Ph3 and Ph6, possibly reflecting a period of metabolic preparation for the future mobilization of reserves, including enzymatic activation and small oscillations prior to more pronounced degradation [38,39,40].

The higher demand for starch during EE and EA observed in Ph3 and Ph6 can be attributed to the increase in transient starch in embryonic tissues, which is shaped by the demand of growing sink tissues. Evidence from rice shows that when photosynthesis is reduced (e.g., during dark growth) starch levels in the embryonic axis decrease along with soluble sugars and the expression and activity of starch biosynthesis enzymes, indicating that transient starch availability responds dynamically to the demands of seedling growth [41,42,43].

During germination, starch can function both as a carbon source, through enzymatic degradation that supplies sugars to the embryo, and as a carbon sink, since reserves in seeds or developing tissues may be transiently re-synthesized according to growth demands. The balance between degradation and synthesis reflects enzymatic and fine genomic regulation that adjusts carbon availability to the physiological stage of the embryo or seedling [44].

Starch hydrolysis depends on the high activity of α-amylase, which converts starch into soluble sugars, essential to sustain respiration and initial seedling growth, and sucrose synthase (SuSy), which cleaves sucrose. These processes become particularly intense soon after germination, when the demand for mobilized carbon is highest [45] α-mylase can be synthesized again during germination, showing a strong increase in activity induced by the gibberellic acid (GA) produced by the embryo, providing sugars that sustain the embryo in the early stages of the germination process [41]. Germination results from the integration of multiple regulatory mechanisms, involving transcriptional and post-transcriptional control, hormonal signaling (GA/ABA), metabolic reprogramming and activation of antioxidant responses necessary to maintain cellular homeostasis during the transition from the quiescent seed to active growth [46].

The decrease and stability of starch in the EA, PD and Eex stages may reflect a reserve maintenance strategy to sustain plant growth until photosynthesis is active, or until other carbon sources are sufficient [47]. The differences observed between phenotypes regarding starch reserves, metabolism, and positive metabolic flux may be associated with a combination of genetic variation in the components of the starch pathway, differences in transcriptional and enzymatic regulation, and physiological plasticity in response to environmental conditions. In *Bertholletia excelsa*, distinct genotypes exhibit dynamic patterns of starch accumulation and mobilization during germination [14].

Allelic variation or differential expression of key genes explains why some phenotypes retain higher starch content in the quiescent seed, while others degrade it rapidly after seed imbibition [48]. Transcriptomic and functional studies show that early activation of α-Amylases promotes rapid starch hydrolysis and provides sugars for respiration and initial growth, which can generate positive flow balances in certain stages [49].

The magnitude of metabolism tends to reflect the intensity of expression of catabolic pathways and enzymatic activity of reserve hydrolysis, in which genotypes with higher transcriptional activity of these pathways generally exhibit higher metabolic activity and a greater capacity for converting reserves into biomass [50]. The presence of transient starch synthesis (re-accumulation in embryonic tissues) has already been documented and depends on the enzymatic control of AGPase, which explains the positive flow balances observed in some phenotypes [51].

In practical terms, the Ph2 phenotype, characterized by a high initial starch content, may be associated with lower enzymatic activation before germination, resulting in greater conservation of reserves. Ph3, which showed higher metabolic activity, possibly expresses more intensely the catabolic pathways responsible for starch degradation. Ph6, exhibiting a higher positive metabolic flux, seems to combine transient synthesis with less degradation at critical stages, culminating in a more favorable final balance. Variations in reserve mobilization rates, as observed among these phenotypes, can directly influence the speed and success of germination [52].

The variations observed in starch content and metabolic flux among Eugenia phenotypes likely reflect differences in the activation of molecular signaling networks that orchestrate germination. During the initial imbibition phase, shifts in the hormonal balance between abscisic acid and gibberellins (ABA/GA) trigger the expression of α-amylases and other hydrolases that catalyze starch degradation, ensuring a continuous supply of soluble sugars vital for early seedling establishment [26,48]. Notably, starch may also act as a transient carbon sink, being resynthesized through the action of enzymes such as ADP-glucose pyrophosphorylase (AGPase). This dynamic turnover suggests that intermediate positive MFI fluxes represent a regulatory strategy to stabilize carbon availability and maintain metabolic homeostasis during crucial developmental transitions [48,51]. In this context, phenotypes such as Ph6, which exhibited transient starch synthesis followed by periods of more intense degradation, may differ in their gibberellin sensitivity and in the enzymatic regulation of starch mobilization and resynthesis pathways, as previously reported for other species. These hypotheses align with existing literature and provide a plausible explanation for the contrasting metabolic fluctuations observed among the phenotypes.

##### Soluble Sugars

The dynamics of soluble sugars among phenotypes reveal contrasting carbon mobilization strategies during germination and initial seedling establishment [53]. The relative stability in sucrose content observed in Ph2 and Ph3, followed by significant increases in later stages, suggests that the seeds maintain metabolic homeostasis during most of their early development. In contrast, Ph6 showed more pronounced oscillations and greater late accumulation of sucrose, reflecting a high metabolic turnover Shi et al. (2024) [45] showed that seeds with sufficient water exhibit high α-amylase and sucrose synthase activity, indicating active metabolism of reserves, which helps maintain sucrose balance and meet developmental demands, supporting seedling growth and vigor in later stages.

The patterns of fructose and glucose reinforce this interpretation. In Ph3, the progressive increase in these sugars in the final stages aligns with the central role of hexoses as respiratory fuel during the transition from the heterotrophic to the autotrophic phase [54]. The intense transient synthesis of fructose in RP for Ph2 and Ph3 suggests that metabolic pulses are associated with emergence, a pattern similar to what is described for *Arabidopsis* seeds, in which hexose peaks precede hypocotyl elongation and cotyledonary expansion [55]. Ph6 demonstrated early transient synthesis and degradation in late stages, accompanied by higher total metabolic activity, indicating greater metabolic flexibility, and this plasticity may allow rapid adjustments between energy demand and substrate availability, a characteristic also observed in species that exhibit vigorous germination and rapid metabolic reorganization [56].

Glucose showed an increasing pattern similar to that of fructose for all the phenotypes, with initial degradation followed by transient synthesis during RP, supporting the idea that the mobilization of reserves operates in cycles of release and redistribution. During seed germination, the glucose and fructose content progressively increases (embryo) as polysaccharide degradation (such as starch) occurs to provide energy [57]. The higher total metabolic activity of glucose in Ph6 confirms its more active pattern of consumption and resynthesis, consistent with the profile observed for sucrose, fructose, and starch.

This set of results indicates that Ph2, Ph3 and Ph6 adopt distinct physiological strategies. Ph2 maintains a higher positive metabolic balance, suggesting a more conservative use of reserves. Ph3 exhibits greater degradative activity, consistent with accelerated metabolism in the initial stages. Ph6 presents the highest overall turnover, alternating between transient synthesis and degradation, enabling continuous growth.

#### 4.2.2. Proteins

The variation observed in soluble protein content among phenotypes is consistent with the pattern already reported in Myrtaceae seeds, characterized by minimal fluctuations during germination. In the present study, phenotypes Ph3 and Ph6 exhibited little significant variation throughout germination, with a more evident increase only in the final stages. This result aligns with observations made for the species *Psidium cattleianum*, in which the variation in soluble proteins during germination was more limited, and the consumption of lipid and carbohydrate reserves was greater [58]. These authors demonstrate that, in Myrtaceae, proteins play a more structural and regulatory role, being mobilized in a smaller proportion when compared to other biochemical reserves.

The behavior of Ph2, which showed consistently reduced levels of soluble protein, peaking only during the EE stage, reinforces this interpretation Melo et al. (2009) [52] evaluated the germination of *E. stipitata* and reported that this species presented low initial protein levels and “little variation in protein content during germination and seedling development” indicating that protein mobilization is minimal throughout the process. Thus, the pattern observed in Ph2, a low initial level and restricted activation at a specific point in germination, is characteristic of species or genotypes whose dominant energy supply does not depend on protein degradation.

In domesticated plants with herbaceous and shrubby habits, such as common beans, the early germination stages are characterized by considerable degradation of protein reserves, while the later stages show intense synthesis activity of these proteins. However, the protein levels throughout this dynamic phase are directly related to the taxonomic group to which the species belongs [59]. In the phenotypes of *E. patrisii*, a similar pattern was observed throughout germination, especially in Ph2 and Ph3, which increased the levels starting from the EE stage.

In contrast, in non-domesticated herbaceous and shrub species from field ecosystems, there is a continuous decline in the concentration of soluble reserve proteins throughout germination as a result of intense degradation, exemplified by *Chloris virgata* and *Kochia scoparia*. This is likely to meet the high demand for amino acids required for rapid growth associated with the life cycle of these plant groups and the reduction in water content [50]. Especially in Ph6, a continuous trend of soluble protein degradation was observed throughout germination, except during the EE and EA stages, in which synthesis was evident.

For herbaceous and shrubby species grown under conditions of full sun, such as *Helianthus annuus*, there is a continuous trend of increased free amino acid synthesis throughout the germination stages [60]. In Ph2 and Ph3 of *E. patrisii*, a similar pattern occurred, with the difference only during PD, in which amino acid degradation was performed, whereas Ph6 oscillated between synthesis and degradation of organic molecules.

Regarding intraspecific variations in seeds of Amazonian fruit trees related to the mobilization of soluble protein reserves and amino acids during germination, recent studies indicate that this represents an important marker of plasticity for the species [13]. In *E. patrisii*, the degradation and synthesis dynamics among phenotypes Ph2, Ph3 and Ph6 suggest the existence of distinct strategies throughout germination and early growth. In *Hevea brasiliensis*, early germination stages showed high synthesis, followed by continuous degradation in subsequent stages, yet still with variation among the different types.

In the genotypes of *Bertholletia excelsa*, the alternation between degradation and synthesis of soluble proteins and amino acids throughout germination has proven to be an effective marker of intraspecific variation, allowing the differentiation of genotypes based on polypeptide dynamics and nitrogen mobilization patterns [14]. Considering this body of evidence, the metabolic behavior of phenotype Ph6, characterized by high initial levels of soluble proteins and pronounced oscillations in amino acid profiles during the final stages, suggests a reserve mobilization strategy associated with greater physiological flexibility throughout germination.

In tropical Amazonian species, patterns characterized by prolonged periods of protein and amino acid synthesis and degradation have been associated with greater metabolic autonomy and enhanced efficiency in the transition to autotrophic growth, as observed in genotypes of *H. brasiliensis* [13] and *B. excelsa* [14]. This alternation between degradation and synthesis ensures a continuous supply of nitrogenous substrates for biosynthetic and respiratory pathways essential to early seedling establishment. Although the present study did not directly evaluate stress responses, the pattern observed in Ph6 is consistent with phenotypes that exhibit greater vigor and physiological resilience, as the sustained availability of mobilizable amino acids and proteins may buffer metabolic costs under fluctuating environmental conditions. Therefore, the distinct mobilization dynamics observed in Ph6 provide functional evidence of enhanced developmental plasticity during the early stages of *E. patrisii* germination.

#### 4.2.3. Total Metabolism

The metabolic patterns observed, including variations in amino acid, soluble protein, simple sugar, and starch profiles, highlight the physiological reprogramming that characterizes the transition from the reserve, dependent heterotrophic metabolism to autotrophic seedling growth [61]. The timing of this mobilization reveals clear differences in reserve-use strategies among the evaluated phenotypes (Figure 5).

In Ph2, the accumulation of soluble proteins and amino acids at the EE stage may reflect the synthesis of early, response proteins or the temporary release of peptides and amino acids through reserve proteolysis, followed by their reutilization or anabolic conversion into cellular proteins required for growth [36]. The pronounced amino acid degradation observed in RP suggests that, at this stage, amino acids are rapidly catabolized to produce energy, via their entry into glycolysis and the TCA cycle, or to synthesize essential secondary metabolites, a pattern commonly reported in metabolomic studies where amino acids and their derivatives exhibit strong correlations with germination rate [62].

In Ph3, the antagonistic pattern between starch and soluble sugars during RE indicates a controlled remobilization process, in which starch serves as a source of monosaccharides that can be consumed both for respiration and for the synthesis of new polymers. Temporal metabolomic studies in seedlings have shown that such alternation is a hallmark of the establishment phase [63].

In Ph6, the early accumulation of glucose and fructose suggests accelerated hydrolysis of carbon reserves, channeling energy toward radicle and hypocotyl emergence. This behavior reflects a rapid transition from a maintenance metabolism to one directed toward central energy, producing, and carbon assimilation pathways, typical of early growth [64].

The PCA revealed distinct strategies in C and N usage. Ph6 relies heavily on starch and amino acids for epicotyl tissue formation, whereas Ph2 and Ph3 primarily utilize simple sugars and total proteins for leaf formation at later stages. At the PD stage, these dynamic reverses: Ph2 and Ph3 intensify the consumption of soluble sugars and proteins to initiate leaf development, while Ph6 supplies both C and N from the onset of this stage. These contrasting patterns suggest that Ph6 prioritizes simple N sources for early foliar formation, while Ph2 and Ph3 prefer simple C sources to sustain epicotyl elongation.

The observed interconversion of C and N throughout early development is consistent with studies demonstrating the progressive reprogramming of energetic pathways during germination [65]. The observed interconversion of C and N throughout early development is consistent with studies demonstrating the progressive reprogramming of energetic pathways during germination [66]. Genotypic differences regulate the preference for simple or complex reserve sources, influencing the efficiency of early growth [67].

Early metabolism is characterized by predominant anaerobic glycolysis due to low mitochondrial activity immediately after imbibition [31]. As mitochondria becomes functionally active, the demand for hexoses increases to sustain aerobic respiration, cell expansion, and tissue differentiation. The late peaks of glucose, fructose, and sucrose observed in *E. patrisii* reflect this transition to a highly respiratory and energetically efficient metabolism.

Reserve mobilization is regulated by the interaction between ABA and GA, which controls the activity of hydrolytic enzymes such as amylases and proteases [68]. Vigorous phenotypes are often associated with more efficient protein mobilization, evidenced by lower residual globulin levels during RP, whereas less vigorous phenotypes display delayed mobilization due to reduced abundance of hydrolytic enzymes.

Differences in MFI, TAI, and NAC indices reinforce their use as valuable tools for phenotypic selection. A negative MFI indicates intense mobilization, often associated with rapid germination and faster growth, whereas a positive MFI suggests reserve retention and potentially higher metabolic resilience. The TAI reflects the accumulated tendency toward reserve conservation or degradation throughout germination, while NAC represents the overall balance between the initial and established seedling stages. Genotypes with positive NAC values tend to be more robust, maintaining greater metabolite availability after germination.

Although phenotypes share similar morphological organization pseudomonocotyledonous embryos with conferruminate cotyledons rich in starch [11], they differ in mobilization efficiency. Highly amyliferous structures, as reported for *E. stipitata* [36], require targeted hydrolysis to sustain germination. In *E. patrisii*, Ph6 exhibits a more flexible mobilization strategy, whereas Ph2 adopts a more conservative one. These differences likely influence vigor, establishment rate, and the transition to autotrophic growth, as suggested by functional diversity models in seed biology [13].

Studies in tropical and forest species further demonstrate that coordinated reserve mobilization and efficient conversion improve early performance and field establishment [13,14]. Collectively, these findings suggest that the metabolic profiles observed during germination may persist and shape subsequent developmental stages. Importantly, the total amount of reserves mobilized does not necessarily determine seedling vigor; rather, vigor depends on how efficiently those reserves are allocated to embryonic growth rather than maintenance metabolism [68]. Thus, phenotypes exhibiting high mobilization, but low conversion efficiency may show reduced growth, whereas those with moderate mobilization yet high conversion efficiency tend to display superior vigor, a pattern consistent with the observations for *E. patrisii*.

Despite the homogeneous management of the mother plants, environmental microvariations can modulate the deposition and mobilization of reserves. However, the consistency of the observed metabolic differences, combined with morphophysiological evidence, indicates that intrinsic phenotypic differences and intraspecific plasticity predominate among the evaluated phenotypes.

## 5. Conclusions

In *Eugenia patrisii*, the biochemical traits of the fruits contributed to phenotypic selection, reflecting maternal investment in reserve compounds that directly influence the physiological performance of the seeds. Fruits of Ph2, with higher carbohydrate and lipid contents, indicated an initial energetic provision; those of Ph6, with elevated protein concentration and total caloric value, suggested a greater supply of amino acids essential for enzymatic synthesis, whereas fruits of Ph3, characterized by higher ash content, evidenced enhanced mineral availability.

These contrasts define quantitative criteria for phenotypic selection, among which the most promising phenotypes combine the following: (i) higher initial carbohydrate levels in the fruit; (ii) greater protein mobilization rate during germination; (iii) a more balanced carbon flux throughout germinative stages. Altogether, the biochemical attributes of fruits and seeds provide an objective basis for identifying phenotypes with superior metabolic efficiency and potential for use in species selection and improvement programs.

Reserve mobilization during germination further revealed contrasting metabolic strategies among phenotypes, with Ph3 and Ph6 standing out for their greater efficiency, stability of biochemical trajectories, and coordinated metabolic fluxes during early development. These metabolic profiles position Ph3 and Ph6 as priority candidates for phenotypic selection and for the identification of biochemical markers relevant to breeding and propagation strategies of this neglected Amazonian fruit species. In this context, the biochemical and nutritional aspects of *E. patrisii* fruits indicate significant potential for cultivating this species. They can also aid in selecting the best genotypes, supporting our hypothesis, and contributing to food security while promoting sustainable development.

## Data Availability

The original contributions presented in this study are included in the article. Further inquiries can be directed to the corresponding authors.
